# Salvaging Salvinorin: From Hallucinogen to Potential Therapeutic through Chemical Synthesis

**DOI:** 10.1021/acscentsci.3c00955

**Published:** 2023-08-10

**Authors:** Bhawyanth Duvvuru, Myles W. Smith

**Affiliations:** UT Southwestern Medical Center, 5323 Harry Hines Blvd., Dallas, Texas 75390, United States

Natural products have served as rich sources of therapeutics dating back to the earliest human civilizations in the form of traditional medicines and more recently as single molecule therapies for a range of diseases. Their diverse biological activities stem from their often complex structures, which can endow natural products with desirable properties relative to synthetic molecules found in typical pharmaceutical libraries.^1^ These same intricate structures can, however, limit chemists’ ability to prepare these compounds de novo—a process termed total synthesis—or restrict derivatives available for biochemical exploration to simple peripheral changes to the natural compound of interest—termed semisynthesis.

**Figure 1:**
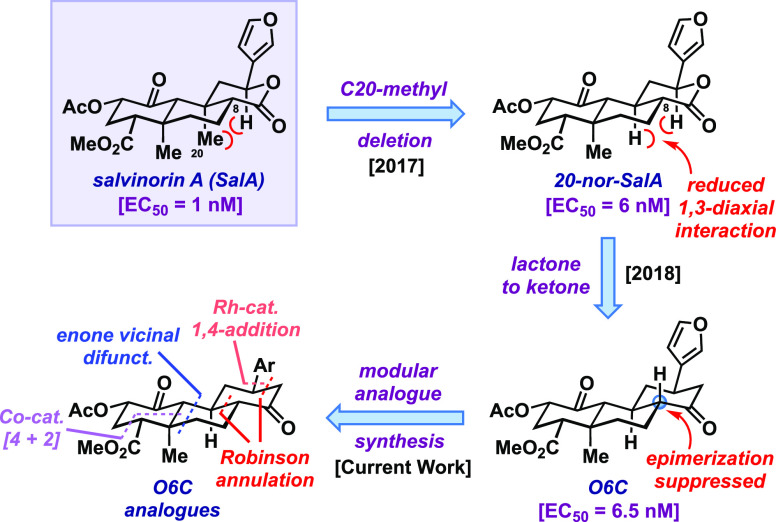
Natural KOR-agonist salvinorin A (SalA), the Shenvi and Bohn groups’ evolving approach toward structurally edited, stabilized, and bioactive SalA analogues, and their modular approach to explore O6C chemical space. [EC_50_ is half-maximal inhibition of forskolin-stimulated cAMP accumulation via KOR (a measure of KOR-agonism)].

One such bioactive natural product that challenges the state of the art in organic synthesis is the polycyclic terpenoid salvinorin A (SalA, Figure 1). SalA is the main psychoactive principle of *Salvia divinorum*, a plant used in traditional Mazatec religion to facilitate visionary states, with SalA itself classified as the most potent naturally occurring hallucinogen ever discovered.^2^ SalA derives its bioactivity from potent and selective agonism of the kappa-opioid receptor (KOR), a target of interest for the development of next-generation analgesics. In this issue of *ACS Central Science*, Shenvi, Bohn, and co-workers showcase a concise and flexible synthetic approach to analogues of SalA that circumvent many of the liabilities associated with the natural compound, while exceeding it in terms of activity, KOR-selectivity, and functional bias.^3^

Traditionally, naturally occurring alkaloids such as morphine have taken center stage among clinically relevant analgesics. Such compounds also interact with opioid receptors—in the case of morphine, most strongly with the mu-opioid receptor (MOR)—to exert their function. Given that receptor selectivity can play a crucial role in the observation of adverse side effects—most poignantly, a high propensity for addiction, contributing to the significant societal burden arising from opioid abuse—it is desirable to find new opioid receptor agonists. KOR-selective compounds, in particular, hold promise as activation of this receptor is not noted to produce addictive effects. In this respect, SalA, which interestingly lacks the basic amine characteristic of KOR agonists,^2^ may offer new opportunities to explore different binding interactions through its oxygenated terpene scaffold.

While several total syntheses of SalA have been issued from the synthetic community, the length of such sequences, their inability to access synthetic analogues for biochemical investigations, and liabilities associated with SalA itself, most notably its propensity to epimerize to a less-active C-8 stereoisomer, have limited its clinical development.^4^ The Shenvi group has previously investigated synthetic SalA structural analogues with the C-20 methyl group excised. This relatively simple change improves the synthetic tractability and serves to stabilize the molecule with respect to C-8 epimerization,^5^ something further reinforced by switching out the ring oxygen atom of the lactone for a methylene unit (O6C), which fully suppressed epimerization (Figure 1).^6^ Of note, neither of these synthetic derivatives would be accessible via semisynthesis. With O6C as a starting point, in the current work the authors fully retool their synthetic approach with a focus on greater brevity, modularity, and the ability to access compounds as single enantiomers.

**Figure 2:**
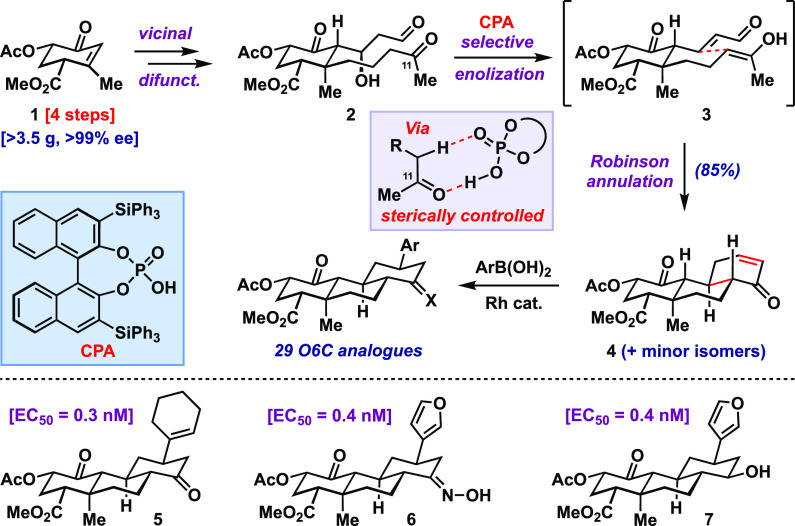
A short enantioselective synthesis, built upon several carefully choreographed transformations, enables the preparation of 29 O6C analogues, several with greater potency than SalA and O6C.

Ultimately, Shenvi et al. arrive at a route whose brevity and simplicity belie the significant effort required for its development. Briefly, their new approach involves the scalable preparation of an enantioenriched oxygenated form of Hagemann’s ester **1** via Co-catalyzed cycloaddition chemistry and an organocatalytic asymmetric Rubottom oxidation (Figure 2). With gram-scale access to enone **1**, the two side chains of **2** that will be laced together in subsequent chemistry to prepare the remaining 6-membered rings can be installed via vicinal difunctionalization. Though this chemistry is well established, here the authors had to resort to fairly esoteric conditions to effect the desired C–C bond formations—yet another reminder that complex natural product-like compounds are often the most challenging of proving grounds for modern synthetic methods. With each of the side chains in place, the authors targeted the construction of the remaining skeletal rings via a variant of the classical Robinson annulation. Standard conditions, however, did not afford the desired tricyclic system (**4**), principally due to the presence of numerous carbonyl moieties within **2**, several of which proved more reactive than the C-11 ketone. To circumvent these issues, the authors leveraged modern chiral phosphoric acid chemistry, where large catalyst substituents can allow for steric effects, rather than α-carbonyl acidity, to guide regioselective enolization to **3**. Indeed, the presence and nature of the bulky groups flanking the reactive phosphoric acid unit appear to be crucial for effecting the desired transformation, creating a defined pocket within which only the more accessible C-11 ketone may bind and be enolized. This feature evokes an analogy to enzyme active sites. Here, this novel application of chiral phosphoric acid catalysis is able to deliver the desired cyclohexenone **4** in excellent yield (85%) along with a few minor stereoisomers. Enone **4** then served as a convenient platform for late-stage diversification through Rh-catalyzed conjugate addition of a variety of carbo- and heterocyclic fragments, as well as reactions of the resulting ketone. All in all, 29 analogues were straightforwardly produced, highlighting the benefit of a modular synthesis design.

Even within this modest set of complex analogues, the authors were able to uncover some promising leads that bested the activity of SalA and their prior lead compound (O6C). For instance, several compounds displayed subnanomolar half-maximal activity (EC_50_, see Figure 2) in assays measuring KOR-agonism. These included a cyclohexenyl congener **5**, and several ketone derivatives including oxime **6** and alcohol **7**. Most strikingly, several of the analogues displayed functional bias toward G-protein signaling over β-arrestin recruitment, a property that holds potential for avoiding undesirable side effects associated with this latter pathway (e.g., sedation, dysphoria).

Though the exact promise of the current set of lead compounds awaits further in vivo studies and determination of their pharmacokinetic properties, including whether they retain SalA’s brain penetrance, the available results are encouraging in terms of the biological plasticity of the SalA scaffold and potential for further structural fine-tuning. The present work underscores the power of modern chemical synthesis to enable deep-seated edits to the structures of complex bioactive natural products without sacrificing modularity or synthetic efficiency. It will be interesting to follow whether the current route proves applicable to SAR studies in distinct regions of the SalA scaffold, or whether this requires a reappraisal of the synthetic strategy. Regardless, as the Shenvi and Bohn groups have amply shown with their evolving work in the salvinorin field, even if a novel synthetic design is called for, this should provide rich opportunities for discovery in chemical synthesis and next-generation analgesic development.
